# *Steinernema Africanum* n. Sp. (Rhabditida, Steinernematidae), a New Entomopathogenic Nematode Species Isolated in the Republic of Rwanda

**DOI:** 10.2478/jofnem-2022-0049

**Published:** 2022-10-26

**Authors:** Ricardo A. R. Machado, Aashaq Hussain Bhat, Joaquín Abolafia, Ebrahim Shokoohi, Patrick Fallet, Ted C. J. Turlings, Eustachio Tarasco, Vladimír Půža, Joelle Kajuga, Xun Yan, Stefan Toepfer

**Affiliations:** 1 Experimental Biology Research Group, Institute of Biology, Faculty of Sciences, University of Neuchâtel, Neuchâtel, Switzerland; 2 Departamento de Biología Animal, Biología Vegetal y Ecología, Universidad de Jaén, Campus ‘Las Lagunillas’, Jaén, Spain; 3 Department of Plant Production, Soil Science and Agricultural Engineering, University of Limpopo, Sovenga, South Africa; 4 Laboratory of Fundamental and Applied Research in Chemical Ecology, Institute of Biology, Faculty of Sciences, University of Neuchâtel, Neuchâtel, Switzerland; 5 CABI Switzerland, Delémont, Switzerland; 6 Department of Soil, Plant and Food Sciences, University of Bari “Aldo Moro”, Bari, Italy; 7 Institute of Entomology, Biology Centre of the Czech Academy of Sciences, České Budějovice, Czech Republic; 8 Department of Crop Innovations and Technology Transfer, Rwanda Agriculture and Animal Resources Development Board, Kigali, Rwanda; 9 Innovative Institute for Plant Health, College of Agriculture and Biology, Zhongkai University of Agriculture and Engineering, Guangzhou, China; 10 MARA-CABI Joint Laboratory for Biosafety, Institute of Plant Protection, Chinese Academy of Agricultural Sciences, Beijing, China

**Keywords:** biocontrol agents, nematode morphology, phylogenetics, species description, taxonomy, *Xenorhabdus*

## Abstract

Alternatives to hazardous insecticides are urgently needed for an environmentally friendly and effective management of insect pests. One such option is the use of entomopathogenic nematodes (EPN). To increase the availability of EPN with potential for biocontrol, we surveyed agricultural soils in the Republic of Rwanda and collected two *Steinernema* isolates. Initial molecular characterization showed that they represent a new species, for which we propose the name *S. africanum* n. sp. To describe this new species, we reconstructed phylogenetic relationships, calculated sequence similarity scores, characterized the nematodes at the morphological level, conducted crossing experiments, and isolated and characterized their symbiotic bacteria. At the molecular level, *S. africanum* n. sp. is closely related to *S. litorale* and *S. weiseri*. At the morphological level, *S. africanum* n. sp. differs from closely related species by the position of the nerve ring and also because the stoma and pharynx region is longer. The first-generation males have ventrally curved spicules with lanceolate manubrium and fusiform gubernaculum and the second-generation males have rounded manubrium and anteriorly hook-like gubernaculum. *Steinernema africanum* n. sp. does not mate or produce fertile progeny with any of the closely related species.

The genus *Steinernema* Travassos, 1927 is one of two major genera of entomopathogenic nematodes (EPNs) used to control insect pests in agriculture. The members of this genus infest and kill numerous insects aided by their symbiotic, entomopathogenic bacteria of the genus *Xenorhabdus*. Together, they constitute a highly valuable pest management tool in sustainable and eco-friendly agriculture (Smart, 1995).

An important step for the use of EPNs in agriculture is the proper description and characterization of the nematode isolates with promising biocontrol traits. In the case of *Steinernema*, there are hundreds of isolates in different laboratories around the globe, which have been assigned to one of the more than 100 *Steinernema* species described so far (Bhat et al., 2020). Many isolates still await being assigned to formal taxonomic studies, and it is very likely that they also represent new, undescribed species.

The species of the genus *Steinernema* are phylogenetically grouped into 12 clades according to the sequences of the internal transcribed spacer (ITS) region of the rRNA (Spiridonov and Subbotin, 2016). There are nine multiple species clades: “*Affine*,” “*Bicornutum*,” “*Cameroonense*,” “*Carpocapsae*,” “*Costaricense*,” “*Feltiae*,” “*Glaseri*,” “*Karii*,” “*Khoisanae*,” “*Kushidai*,” “*Longicaudum*,” and “*Monticolum*”; and three monospecies clades: *S. neocurtillae*, *S. unicornum*, and *S. rarum* (Spiridonov and Subbotin, 2016). The “*feltiae*-clade” currently contains at least 16 species, and many of them are closely related to the novel *S. africanum* n. sp., described in this study (Spiridonov and Subbotin, 2016). These are *S. citrae* Stokwe et al., 2011, *S. feltiae* (Filipjev, 1934) Wouts, Mráček, Gerdin & Bedding, 1982; *S. hebeiense* Chen et al., 2006; *S. ichnusae* Tarasco et al., 2008; *S. litorale* Yoshida, 2005; *S. nguyeni* Malan et al., 2016; and *S. weiseri* Mráček et al., 2003.

During a survey of agricultural soils in the Republic of Rwanda in 2014, two nematode isolates, *Steinernema* sp. RW14-M-C2b-1 and RW14-M-C2a-3, were recovered (Yan et al., 2016). Initial molecular characterization suggested that they represent a new species. In this study, we describe this new EPN species through the reconstruction of phylogenetic relationships based on nuclear and mitochondrial genes, sequence similarity calculations, morphological and morphometric characterizations, self-crossing and cross-hybridization experiments, and the isolation and characterization of their symbiotic bacteria. This study contributes to a better understanding of the biodiversity and phylogenetic relationships of an important group of biological control agents, which is essential for the establishment of biocontrol programs in sustainable and eco-friendly agriculture.

## Materials and Methods

### Nematode origin

*Steinernema africanum* n. sp. RW14-M-C2b-1 and RW14-M-C2a-3 nematodes were isolated from soils of a banana, pumpkin, and sorghum intercrop in a valley of the Republic of Rwanda (GPS coordinates: 1°28´11.1"S 29°41´36.2"E; 1,865 m. s. n. m.) (Yan et al., 2016). Nematode isolation was achieved by baiting mixed soil samples with *Galleria mellonella* (Lepidoptera: Pyralidae) larvae, and placing the infected larvae in White traps (White, 1927).

### Nematode morphological and morphometrical characterization, light and scanning electron microscopy

First- and second-generation adult nematodes were obtained by dissecting infested *G. mellonella* cadavers in Ringer’s solution after 5 d to 6 d and 8 d to 9 d of post infestation, respectively. Infective juveniles (IJs) were collected after their emergence from *G. mellonella* larvae in White traps (White, 1927). Nematodes were killed with water at 60°C, fixed in 4% formalin solution (4 mL formaldehyde, 1 mL glycerol, and 95 mL ddH_2_O) and transferred to anhydrous glycerin by the Seinhorst method (Seinhorst, 1959). Nematodes were then mounted on permanent glass slides with a thicker layer of paraffin wax to prevent the flattening of the nematodes (Grisse, 1969). Morphological measurements were taken using the Olympus BX51 software built into the ZEISS Axio Lab. A1 light microscope (Carl Zeiss Microscopy GmbH, Jena, Germany). Fifteen specimens of *S. africanum* n. sp. RW14-M-C2b-1 at each developmental stage were measured. To obtain light microscopy (LM) and scanning electron microscopy (SEM) photographs, specimens were processed following techniques described in detail by Abolafia (2022). Briefly, the nematodes, fixed in 4% formalin solution, were processed to anhydrous glycerin with Siddiqi’s method using lactophenol-glycerin solutions (Siddiqi, 1964). Then, the nematodes were permanently mounted on glass microscope slides using the glycerin-paraffin method (Maeseneer and d’Herde, 1963; Siddiqi, 1964). Light microscopy photographs were taken using a Nikon Eclipse 80i microscope (Olympus, Tokyo, Japan) equipped with differential interference contrast optics (DIC) and a Nikon Digital Sight DS-U1 camera. For scanning electron microscopy, nematodes preserved in glycerin were taken from the permanent microscope slides by removing the cover glass, re-hydrated in distilled water, dehydrated in a graded ethanol-acetone series, critical point dried with liquid CO_2_, mounted on SEM stubs with copper tape, coated with gold in a sputter coater, and finally observed with a Zeiss Merlin microscope (5 kV) (Zeiss, Oberkochen, Germany) (Abolafia, 2015). Light microscopy micrographs, obtained at different levels for each structure, were processed and combined using AdobeÒ PhotoshopÒCS (Microsoft Corporation, Redmond, WA). Morphological characters of closely related species were taken from the original publications (Hunt and Nguyen, 2016).

### Self-crossing and cross-hybridization experiments

Self-crossing and cross-hybridization experiments were conducted as described by Kaya and Stock (1997) with some minor modifications (Kaya and Stock, 1997). Briefly, drops of hemolymph obtained from surface-sterilized *G. mellonella* larvae were placed in sterile Petri dishes (35 mm x 10 mm). A few micrograms of phenylthiourea were added to hemolymph drops to prevent melanization. Then 40 to 60 surface-sterilized juvenile nematodes (IJs) were added to the hemolymph drops. Nematodes were surface sterilized by immersing them in 0.1% NaOCl, and then washed thrice with autoclaved double distilled water. Then, Petri dishes were wrapped in moistened paper tissue and kept in plastic bags at 25°C. Petri plates were observed daily until IJs developed into adults. Then, male and female adults were separated by observing them under a light microscope. For self-crossing experiments, three males and three females of the same species were transferred to fresh hemolymph drops as described above. For cross-hybridization experiments, three males and three females of different species were transferred to fresh hemolymph drops as described above. Females without males were also included to confirm their virginity. Petri plates were observed daily to determine the production of offspring. For each crossing type, 10 independent Petri plates were included. Experiments were conducted twice under the same conditions. The following species were included in these experiments: *Steinernema africanum* n. sp. RW14-M-C2b-1 and RW14-M-C2a-3, *S. feltiae* Jakub, *S. ichnusae* Sardinia, *S. litorale* Aichi, and *S. weiseri* 1025 (Yoshida, 2004; Tarasco et al., 2008; Půža et al., 2021).

### Nematode molecular characterization and phylogenetic relationships

Genomic DNA from about 20 females was extracted using the genomic DNA isolation kit from QIAamp DNA Mini Kit (Qiagen, Valencia, CA) following the manufacturer’s instructions. The following genes/ genomic regions were amplified by polymerase chain reaction (PCR): the D2–D3 expansion segments of the 28S rRNA, the ITS region of the rRNA, the mitochondrial 12S rRNA, and the cytochrome oxidase subunit I (COI). To amplify the ITS rRNA, the following primers were used: 18S (5´-TTGATTACGTCCCTGCCC TTT-3´) and 26S (5´-TTTCACTCGCCGTTACTAAGG-3´) (Joyce et al. 1994). To amplify the D2–D3 region, the following primers were used: D2F (5´- CCTTAGTAAC GGCGAGTGAAA-3´) and 536 (5´-CAGCTATCCTGA GGAAAC-3´) (Subbotin et al., 2006). To amplify the 12S mitochondrial rRNA gene, primers 505F: 5´-GTTCCAG AATAATCGGCTAGAC-3´ and 506R: 5´-TCTACTTTACT ACAACTTACTCCCC-3´ were used (Nadler et al., 2006). Primers LCO-1490 (5´-GGTCAACAAATCATAAA GATATTGG-3´) and HCO-2198 (5´-TAAACTTCAGGGT GACCAAAAAATCA-3´) were used to amplify the COI (Folmer et al., 1994). PCR reactions consisted of 12.5 μL of DreamTaq Green PCR Master Mix (Thermo Scientific, Waltham, MA USA), 0.5 μL of each forward and reverse primers at 10 μM, 1 μL of genomic DNA, and 10.5 μL of nuclease free distilled water. The PCR reactions were performed using a thermocycler with the following settings. For ITS and D2–D3: 1 cycle of 5 min at 94°C followed by 40 cycles of 30 sec at 94°C, 30 sec at 50°C, 1 min 30 sec at 72°C, and by a single final elongation step at 72°C for 10 min. For the 12S gene, the PCR protocol included initial denaturation at 94°C for 3 min, followed by 30 cycles of 94°C for 30 sec, 50°C for 30 sec, and 72°C for 45 sec, followed by a final extension at 72°C for 15 min. For the COI gene, the PCR program was as follows: 1 cycle of 94°C for 2 min, followed by 37 cycles of 94°C for 30 sec, 51°C for 45 sec, 72°C for 2 min, and a final extension at 72°C for 12 min. PCR was followed by electrophoresis (45 min, 100 V) of 10 μL of PCR products in a 1% TBA (Tris– boric acid–EDTA) buffered agarose gel stained with SYBR Safe DNA Gel Stain (Invitrogen, Carlsbad, CA). PCR products were purified using QIAquick PCR Purification Kit (Qiagen, Valencia, CA) and sequenced using reverse and forward primers by Sanger sequencing (Microsynth AG, Balgach, Switzerland). Obtained sequences were manually curated and trimmed and deposited in the National Center for Biotechnology Information (NCBI) under the accession numbers given on the phylogenetic trees. To obtain genomic sequences of nematodes that belong to all the validly described species of the “*feltiae-*clade,” we searched the database of the NCBI using the Basic Local Alignment Search Tool (Altschul et al. 1990). The resulting sequences were used to reconstruct phylogenetic relationships by the maximum likelihood method based on the following nucleotide substitution models: Hasegawa–Kishino– Yano model (HKY + G) (ITS), General Time Reversible model (GTR + G + I) (COI), Kimura 2-parameter (K2 + G + I) (D2–D3), and Tamura 3-parameter model (T92) (12S) (Kimura, 1980; Hasegawa et al., 1985; Tamura, 1992; Nei and Kumar, 2000). To select the best substitution models, best–fit nucleotide substitution model analyses were carried out in MEGA 7 (Kumar et al., 2016). Sequences were aligned with MUSCLE (v3.8.31) (Edgar, 2004). The trees with the highest log likelihood are shown. The percentage of trees in which the associated taxa clustered together is shown next to the branches. Initial tree(s) for the heuristic search were obtained automatically by applying Neighbor– Join and BioNJ algorithms to a matrix of pairwise distances estimated using the maximum composite likelihood (MCL) approach, and then selecting the topology with superior log likelihood value. In some cases, a discrete Gamma distribution (+G) was used to model evolutionary rate differences among sites and the rate variation model allowed for some sites to be evolutionarily (+I). The trees are drawn to scale, with branch lengths measured in the number of substitutions per site. Graphical representation and edition of the phylogenetic trees were performed with Interactive Tree of Life (v3.5.1) (Chevenet et al., 2006; Letunic and Bork, 2016).

### Symbiotic relationships

The entomopathogenic *Xenorhabdus* bacteria associated with *S. africanum* n. sp. RW14-M-C2b-1 nematodes were isolated as described (Machado et al., 2018, 2019). To establish their taxonomic identities, we reconstructed phylogenetic relationships based on whole genome sequences of the isolated bacteria and all the different species of the genus *Xenorhabdus*. Genomic sequences were obtained as described (Machado et al., 2021b, 2021c). Genome sequences were deposited in the National Centre for Biotechnology Information. Accession numbers are listed in Table S1 in Supplementary Material. Phylogenetic relationships were reconstructed based on the assembled genomes and the genome sequences of all validly published species of the genus with publicly available genome sequences as described (Machado et al., 2021a). Whole genome sequence similarities were calculated by the digital DNA–DNA hybridization (dDDH) method using the recommended formula 2 of the genome-to-genome distance calculator (GGDC) web service of the Deutsche Sammlung von Mikroorganismen und Zellkulturen GmbH (DSMZ) (Auch et al., 2010a, 2010b; Meier-Kolthoff et al., 2013, 2014).

### Life cycle

The life cycle of *S. africanum* n. sp. was studied by infesting wax moth larvae (*G. mellonella*) with either 50 or 150 *S. africanum* n. sp. RW14-M-C2b-1 IJs per larva (*n* = 30). Larvae were individually placed in Petri plates lined with a sheet of moist filter paper and incubated at 24°C. Upon infection, a few cadavers were dissected daily to collect and observe the number of nematodes at each developmental stage.

## Results and Discussion

Two populations of *Steinernema* nematodes, RW14-M-C2b-1 and RW14-M-C2a-3, were isolated from agricultural soils in the Republic of Rwanda (Yan et al., 2016). Initial molecular characterization showed that they are identical, belong to the “*feltiae-*clade,” are closely related to *S. feltiae*, *S. citrae*, *S. litorale*, *S. nguyeni*, and *S. weiseri*, and represent a new species, which we named *S. africanum* n. sp. To describe this new species, we compared it with other closely related species at the molecular and morphological level, and conducted cross-hybridization and self-crossing experiments. As both populations are identical at the molecular level, we selected RW14-M-C2b-1 for detailed morphological characterization.

### *Steinernema africanum* n. sp

**Figure 1:**
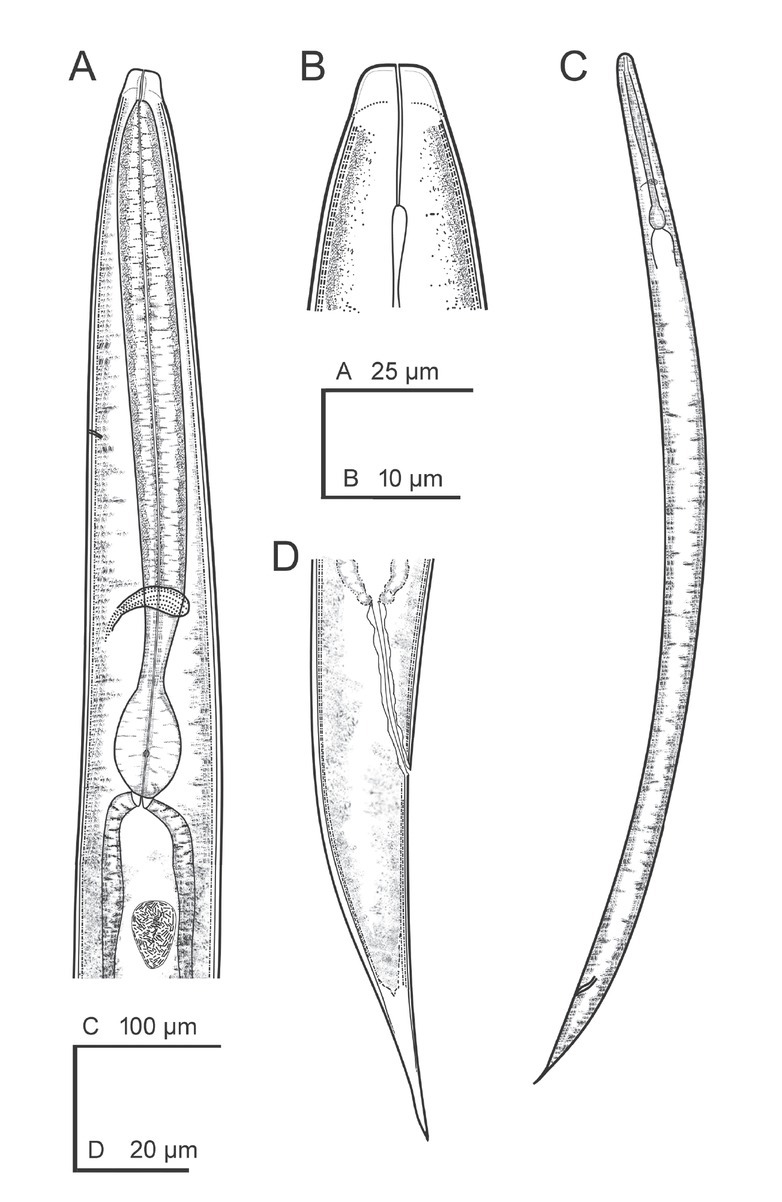
Line drawings of *Steinernema africanum* n. sp. IJ. **(A)** Stoma and pharynx region. **(B)** Anterior end. **(C)** Entire IJ. **(D)** Posterior end. IJ, infective juvenile.

**Figure 2:**
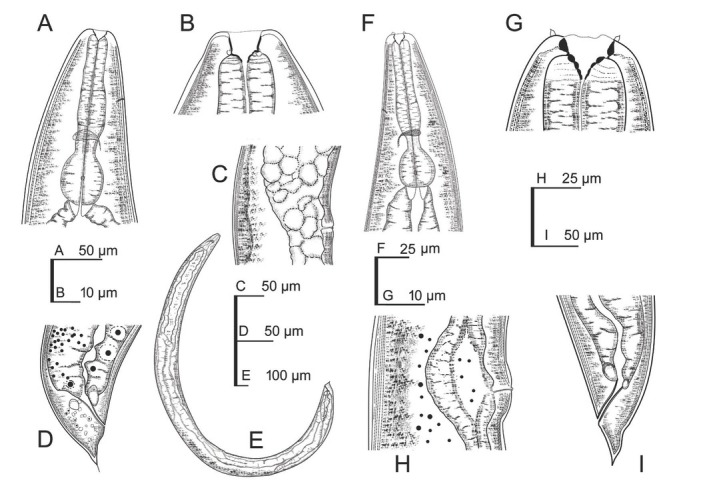
Line drawings of first- and second-generation *Steinernema africanum* n. sp. females. **(A–E)** First-generation female: **(A)** Stoma and pharynx region; **(B)** Lip region and stoma; **(C)** Vagina region; **(D)** Posterior end; **(E)** Entire female. **(F–I)** Second-generation female: **(F)** Stoma and pharynx region; **(G)** Lip region and stoma; **(H)** Vagina region; **(I)** Posterior end.

**Figure 3:**
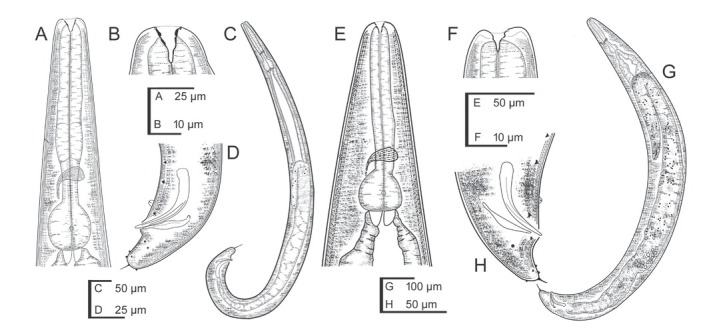
Line drawings of first- and second-generation *Steinernema africanum* n. sp. males. **(A–D)** First-generation male: **(A)** Stoma and pharynx region; **(B)** Lip region and stoma; **(C)** Entire male; **(D)** Posterior end. **(E–H)** Second-generation male: **(E)** Stoma and pharynx region; **(F)** Lip region and stoma; **(G)** Entire male; **(H)** Posterior end.

**Figure 4:**
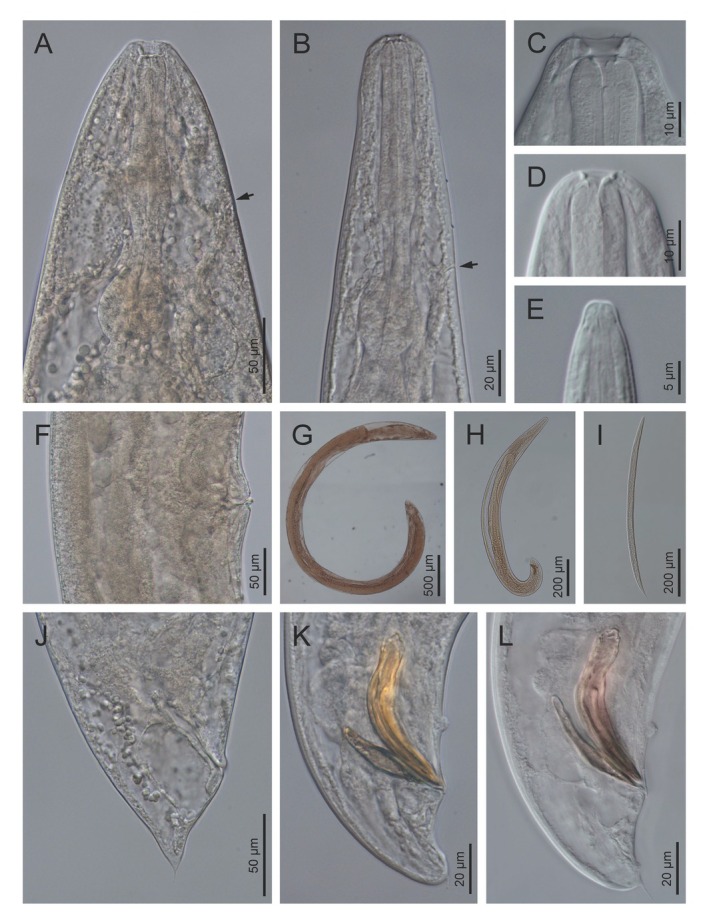
Light microscope micrographs of first-generation adults and IJ of *Steinernema africanum* n. sp. **(A, B)** Stoma and pharynx region of female and male, respectively. **(C–E)** Lip region and stoma of female, male and IJ, respectively. **(F)** Vagina region. **(G–I)** Entire female, male and IJ, respectively. **(J)** Posterior end of a female. **(K, L)** Posterior end of males. Pictures are in right lateral view. IJ, infective juvenile.

**Figure 5:**
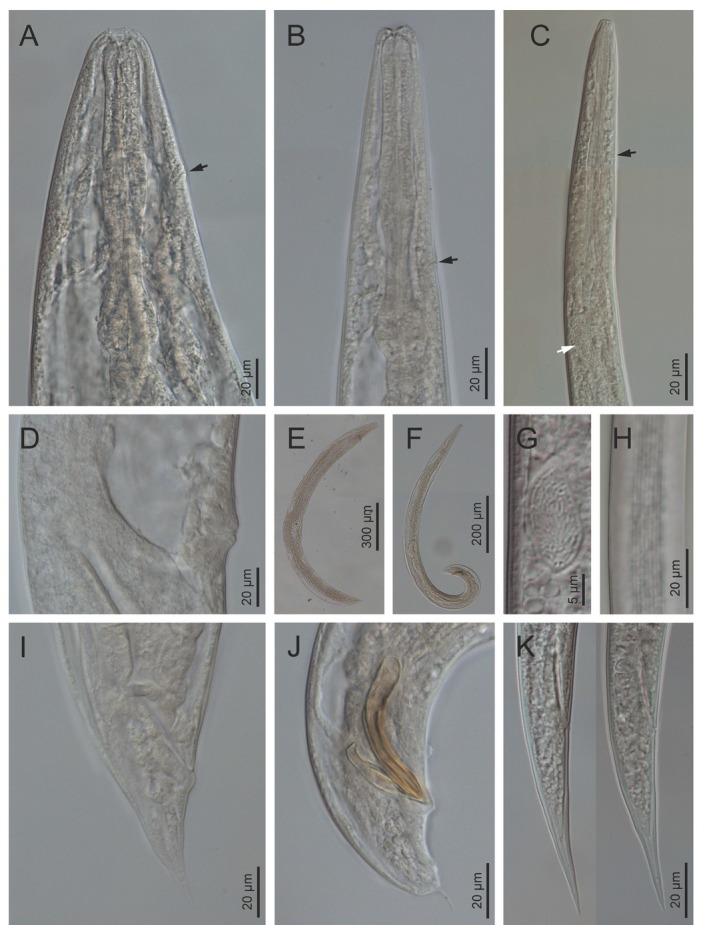
Light microscope micrographs of second-generation adults and IJ of *Steinernema africanum* n. sp. **(A–C)** Stoma and pharynx region of female, male and IJ, respectively. Black arrow pointing the EP, white arrow pointing the bacteria sac. **(D)** Vagina region. **(E, F)** Entire female and male, respectively. **(G, H)** IJ bacterial sac and lateral field, respectively. **(I–K)** Posterior end of female, male, and IJ, respectively. Pictures are in right lateral view. EP, excretory pore; IJ, infective juvenile.

**Figure 6:**
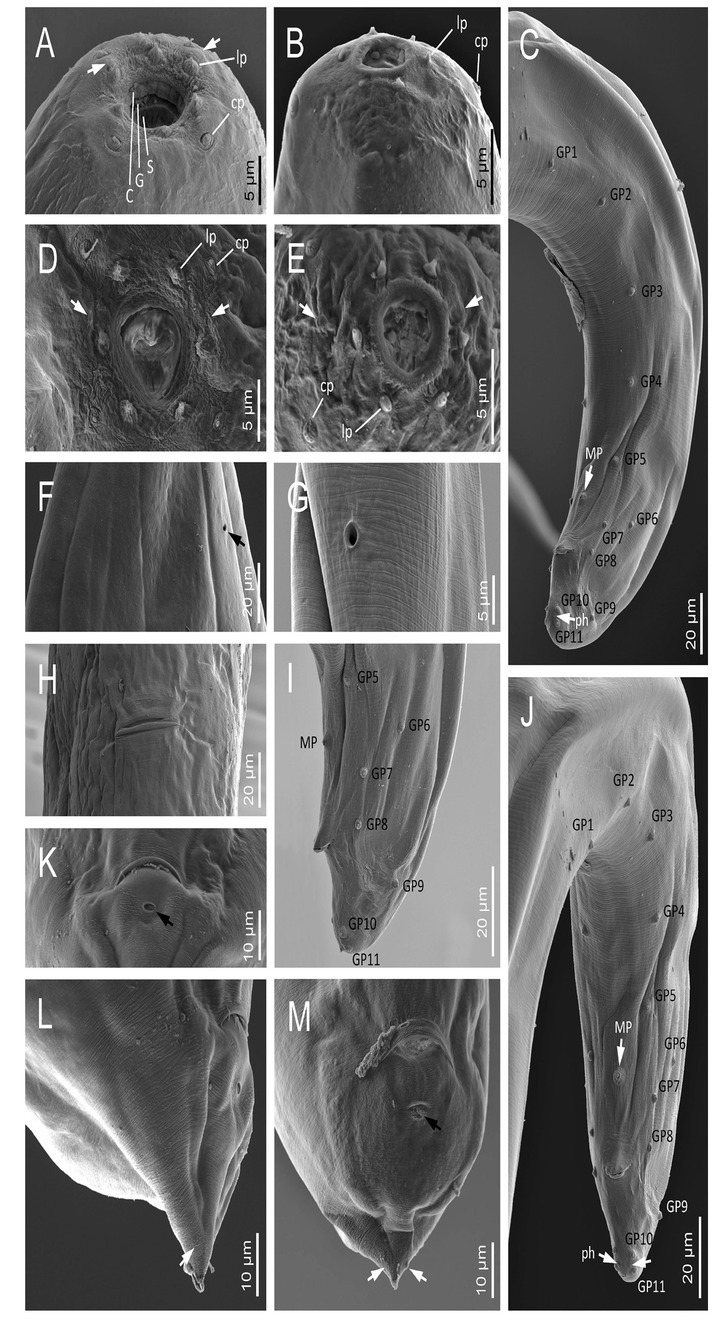
Scanning electron microscope micrographs of first-generation adults of *Steinernema africanum* n. sp. **(A, B)** Lip region of a female and a male, respectively, in lateral view. Arrows pointing the amphids (C: cheilostom, G: gymnostom, S: stegostom, cp: cephalic papillae, and lp: labial papillae). **(C)** Male posterior end in sub-ventral view – GP, MP, ph. **(D, E)** Lip region of a female and a male, respectively, in frontal view. **(F, G)** EP of a female and a male, respectively. **(H)** Vulva. **(I)** Male posterior end in sub-ventral view – GP, MP, ph. **(J)** Male posterior end in sub-ventral view – GP, MP. **(K)** Female anus. Arrow pointing a post-anal pore. **(L, M)** Female tail in lateral and ventral views, respectively. Black arrow pointing a post-anal pore, white arrows pointing the phasmids. EP, excretory pore; GP, genital papillae; MP, mid-ventral papilla; ph, phasmid.

**Figure 7:**
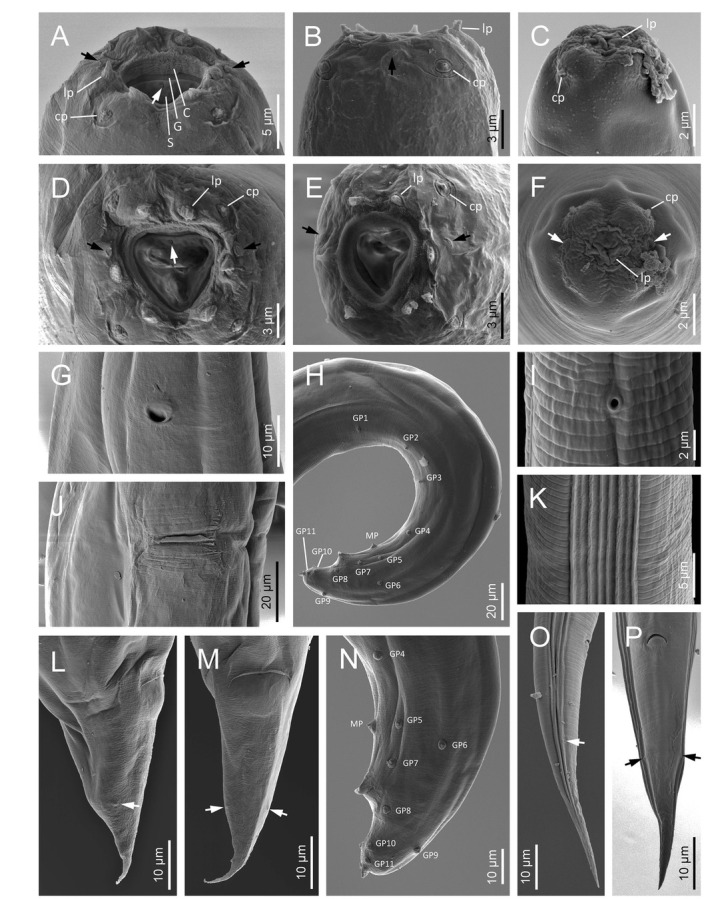
Scanning electron microscope micrographs of second-generation adults and IJ of *Steinernema africanum* n. sp. **(A–C)** Lip region of female, male, and IJ in lateral view, respectively. Arrows pointing the amphids (C: cheilostom, G: gymnostom, S: stegostom, cp: cephalic papillae, lp: labial papillae). **(D–F)** Lip region of female, male, and IJ in frontal view, respectively. Black arrows point to the amphids, white arrow points to the dorsal gland opening. **(G)** EP of female. **(H)** Male posterior region in lateral view – GP, MP, ph. **(I)** EP of IJ. **(J)** Vulva. **(K)** IJ lateral field. **(L, M)** Female tail (arrows pointing the phasmids). **(N)** Male posterior region in lateral view. **(O, P)** IJ tail in lateral and ventral views, respectively. Arrows pointing the phasmids. EP, excretory pore; GP, genital papillae; MP, mid-ventral papilla; ph, phasmid.

(Figures 1–7 and Tables 1–4)

**Table 1:** Morphometrics of IJs and adult generations of *Steinernema africanum* n. sp.

Characters	Male first generation		Male second generation	Female first generation	Female second generation	Infective juvenile
Specimen type	Holotype	Paratype	Paratype	Paratype	Paratype	Paratype
n	1	15	15	15	15	15
Body length (L)	1,300	1,202.2 ± 141.4 (977‒1,400)	955.4 ±92.8 (831‒1,213)	3,319.1 ± 763.2(2,469‒5,033)	1,620.0 ±487.6 (924‒2,311)	750.9 ± 39.8 (690‒802)
a (L/BD)	9.9	10.3 ±0.9 (9.1‒11.6)	17.0 ±1.6 (14.3‒19.0)	18.0 ±3.9 (13.2‒27.0)	13.5 ±1.0 (12.1‒15.7)	28.0 ± 2.4 (23.2‒30.4)
b (L/NL)	8.8	8.4 ± 0.8 (7.2‒9.6)	7.3 ± 0.6 (6.6‒8.5)	17.4 ±3.7 (12.8‒23.9)	10.1 ±2.1 (7.2‒12.9)	4.9 ± 0.5 (4.3‒6.3)
c (L/TL)	28.3	29.0 ± 3.3 (24.5‒33.9)	29.7 ± 2.9 (25.8‒35.7)	74.1 ± 17.6 (50.6‒103.9)	33.5 ±11.7 (16.5‒50.9)	11.9 ± 1.3(10.3‒14.9)
c' (TL/ABW)	0.9	1.0 ±0.1 (0.9‒1.1)	1.0 ±0.1 (0.7‒1.1)	0.9 ±0.1 (0.7‒1.0)	1.5 ±0.3(1.2‒2.3)	3.7 ± 0.4 (2.9‒4.2)
T(MR/L × 100),	94	87.3 ± 11.8 (65‒99)	71.6 ± 13.4 (58‒96)	52.0 ± 2.7 (50‒57)	56.0 ± 1.9 (53‒58)	‒
V (VA/L × 100)						
Lip region diameter	21	19.5 ±3.2(13‒25)	15.1 ±1.6 (11‒17)	24.9 ±3.3(19‒31)	22.3 ±6.5 (14‒34)	6.1 ± 0.7 (5‒7)
Stoma length	9	7.5 ±2.1 (5‒11)	6.7 ± 1.2 (5‒9)	12.8 ± 2.9 (8‒19)	11.1 ± 2.4 (8‒16)	17.6 ± 4.4 (10‒23)
Corpus length	84	83.5 ± 5.2 (72‒90)	69.9 ± 3.3 (65‒76)	91.9 ± 8.1 (77‒108)	83.0 ± 14.0 (62‒101)	85.5 ± 8.1 (72‒97)
Isthmus length	26	20.8 ± 3.3 (14‒26)	22.4 ± 2.7 (18‒28)	27.6 ± 5.6 (21‒40)	23.8 ± 7.1 (11‒35)	27.6 ± 5.4 (19‒37)
Bulb length	28	29.2 ± 3.7 (25‒38)	26.9 ± 2.6 (22‒32)	45.1 ± 6.6 (37‒58)	33.3 ± 4.7 (24‒42)	22.8 ± 2.0 (18‒26)
Cardia length	11	8.7 ±1.5 (6‒11)	5.8 ±1.8 (4‒11)	11.5 ± 3.1 (8‒19)	7.7 ± 2.7 (4‒12)	‒
NR to anterior	95	91.5 ± 8.1 (79‒104)	83.5 ± 7.6 (74‒101)	105.4 ± 14.9 (79‒130)	96.4 ± 19.8 (69‒124)	117.7 ± 11.5 (87‒132)
EP to anterior	105	93.4 ± 12.4 (69‒109)	91.2 ± 6.7 (83‒104)	89.4 ± 14.3 (67‒111)	83.6 ± 11.6 (61‒104)	59.3 ± 4.2 (54‒68)
PL	138	134.7 ± 5.4 (123‒142)	123.5 ± 5.6 (117‒135)	169.6 ± 8.8 (158‒188)	140.9 ± 19.7 (113‒167)	137.2 ± 9.2 (113‒153)
NL(Stoma+PL)	147	142.5 ± 4.4 (132‒147)	131.1 ±5.6 (124‒143)	185.3 ± 7.9 (170‒201)	152.6 ± 21.6 (122‒181)	154.4 ± 12.0 (123‒167)
BD at neck base	73	65.7 ± 10.8 (41‒76)	44.7 ± 11.4 (37‒73)	124.4 ± 9.3 (113‒153)	77.5 ± 17.4 (47‒105)	26.1 ±1.6(21‒29)
BD at midbody	131	114.8 ± 17.9 (65‒131)	54.1 ± 5.2 (39‒59)	181.0 ± 14.8 (154‒208)	114.7 ± 37.3 (59‒167)	26.9 ± 1.6 (25‒32)
Anal BD (ABW)	50	41.9 ± 5.5 (33‒50)	34.8 ± 5.7 (30‒48)	52.4 ± 8.2 (37‒70)	33.9 ± 5.8 (26‒43)	17.2 ± 1.1 (16‒20)
Vagina length	‒	‒	‒	20.8 ± 6.3 (11‒33)	24.2 ± 11.9 (10‒49)	‒
VA end	‒	‒	‒	1,767.1 ± 445.4 (1,284‒2,411)	905.9 ± 266.0 (516‒1,217)	‒
Rectum length (RL)	‒	‒	‒	46.4 ± 8.8 (33‒63)	37.3 ± 14.3 (20‒64)	28.3 ± 3.9 (23‒38)
Tail length (TL)	46	41.1 ± 4.0 (34‒46)	33.9 ± 4.8 (28‒46)	44.6 ± 5.9 (35‒55)	49.7 ±5.7 (40‒60)	64.0 ± 7.1 (52‒72)
Tail hyaline length (H)	‒	‒	‒	7.4 ± 2.6 (4‒11)	12.2 ± 3.8 (6‒19)	22.5 ± 3.7 (17‒27)
SL	*72*	71.1 ± 3.6 (65‒76)	59.5 ± 4.8 (53‒68)	‒	‒	‒
GL	42	42.5 ± 4.2 (32‒49)	32.6 ± 5.7 (25‒46)	‒	‒	‒
Stoma length/lip region width	0.4	0.4 ± 0.1 (0.2‒0.7)	0.4 ± 0.1 (0.3‒0.5)	0.5 ± 0.1 (0.3‒0.6)	0.5 ± 0.1 (0.3‒0.7)	2.9 ± 0.6 (1.5‒3.7)
NR% (NR/NL × 100)	65	63.9 ± 4.9 (56‒71)	63.7 ± 3.5 (60‒71)	56.9 ± 7.9 (43‒65)	63.2 ± 8.5 (43‒69)	76.3 ± 5.8 (69‒90)
D % (EP/NL × 100)	71	65.1 ± 7.2 (52‒74)	69.6 ± 2.7 (67‒75)	48.3 ± 8.4 (36‒62)	55.5 ± 9.7 (49‒79)	38.1 ± 2.7 (34‒46)
E% (EP/TL × 100)	228	224.6 ± 22.7 (197‒261)	284.3 ± 34.5 (243‒345)	204.8 ± 44.9 (132‒281)	170.9 ± 33.4 (122‒221)	94.7 ± 17.4 (79‒129)
Rectum % (RL/ABD × 100)	‒	‒	‒	90.4 ± 19.7 (58‒112)	108.1 ±29.3 (74‒163)	164.3 ± 17.1 (128‒211)
H % (H/TL × 100)	‒	‒	‒	15.7 ± 5.1 (8‒20)	21.6 ± 11.7 (12‒40)	35.2 ± 3.4 (28‒39)
SW% (SL/ABD × 100)	144.0	172.3 ± 21.3 (144‒197)	177.2 ± 19.8(142‒217)	‒	‒	‒
GS % (GL/SL × 100)	58	59.9 ± 5.9 (49‒68)	54.7 ± 7.1 (46‒68)	‒	‒	‒
Male reproductive system (MR)	1,218	1,044.3 ± 240.9 (639‒1,371)	692.9 ± 172.4 (553‒1,166)			
Testis reflexion	281	280.2 ± 66.3 (116‒352)	159.7 ± 64.6 (89‒273)	‒	‒	-
Mucron	5.1	4.8 ± 0.7 (4.0-5.3)	7.3 ± 2.5 (5-10)	4.4 ± 0.7 (3.7-5.0)	Absent	-

All characters are in μm (except ratios and percentages) and given as mean ± s.d. (range). BD, body diameter; EP, excretory pore; GL, gubernaculum length; IJs, infective juveniles; NL, neck length; NR, nerve ring; PL, pharynx length; SL, spicule length; VA, vulva-anterior end.

### First-generation male

Body slender, ventrally curved posteriorly, C- or J-shaped when heat-killed. Cuticle with transversal incisures scarcely marked, with annuli appearing slightly visible. Lateral fields and phasmids inconspicuous under LM. Lip region truncate to slightly round, continuous with body. Six lips amalgamated, with one acute labial papilla and one low cephalic papilla each, except lateral lips. Amphidial apertures small, located at lateral lips posterior to lateral labial papillae. Stoma shallow, funnel-shaped, short and wide, with inconspicuous sclerotized walls; cheilostom short with small rhabdia; gymnostom scarcely developed with minute rhabdia stegostom robust with funnel-shaped lumen and walls with minute rhabdia. Deirids inconspicuous. Pharynx muscular with a cylindrical procorpus, a slightly swollen and non-valvate metacorpus, narrower isthmus and basal bulb spheroid with reduced valves. Nerve ring (NR) usually located about mid-isthmus level or on the anterior part of the basal bulb. Secretory-EP opening circular, located posterior to NR, close to isthmus-basal bulb junction. Cardia prominent, conoid. Intestine tubular without differentiations. Reproductive system monorchic, ventrally reflexed. Spicules paired, symmetrical, ventrally curved with manubrium rhomboidal, calamus narrower and lamina ventrad curved at anterior part, bearing two longitudinal ribs, and ending in a blunt terminus, with scarcely developed velum not reaching the spicule tip, without rostrum or retinaculum. Gubernaculum fusiform with elongate tip, about one-half of the length of spicules. Tail conoid with rounded terminus bearing a fine mucron. Bursa absent. There are 23 GP (11 pairs and one single) arranged as follows: five pairs sub-ventral precloacal, one pair lateral precloacal, one single mid-ventral papilla, two pairs sub-ventral ad-cloacal, one pair subdorsal post-cloacal, and two pairs of terminal papillae. Phasmids terminal, located between the last pair of GP.

### Second-generation male

General morphology similar to that of first-generation males, but smaller in size and slenderer. Tail with mucron robust, dorsally curved. Spicules ventrally curved, with manubrium rounded, calamus slightly narrower than manubrium, and lamina ventrally curved at anterior part, lanceolate posterior part with finely rounded tip, reduced ventral velum, and two longitudinal lateral ribs. Gubernaculum slenderer than that of first-generation male, with manubrium ventrad bent, corpus robust, and narrow and slender terminus. Genital papillae and phasmids with arrangement similar to that in first-generation male.

### First-generation female

Body C-shaped when heat-relaxed and fixed. Cuticle with transversal incisures marked, appearing poorly visible annuli. Lateral fields not observed. Deirids inconspicuous, difficult to observe even under SEM. Labial region rounded, continuous with the adjacent part of body. Stoma and pharynx region similar to males. Excretory pore located at level of the metacorpus-isthmus junction. Nerve ring surrounding the isthmus. Reproductive system didelphic, amphidelphic. Ovaries reflexed in dorsal position; oviducts well developed with glandular spermatheca, and uteri tubular with numerous uterine eggs; vagina short, with muscular walls; vulva protruding, in the form of transverse slit located slightly post-equatorial with lips slightly protruding, asymmetrical, with small epiptygma. Rectum 0.6 to 1.1 times the BD, with three rectal glands. Tail conoid, shorter than body anal diameter, with terminus bearing a fine mucron. Phasmids located at anterior part of tail, at 23% to 34% of tail length.

### Second-generation female

Similar to first-generation female but smaller. Tail conoid, longer than the first-generation female, lacking mucron. Phasmid located at the posterior part of the tail, at 58% to 59% of tail length.

### Third-stage infective juvenile

Body straight or slightly curved when heat-killed, tapering gradually from the base of pharynx to the anterior end and from anus to the distal end. Cuticle with transverse incisures, appearing well-developed annuli. Lateral fields begin as a single line close to the anterior end, increasing to eight ridges, posteriorly gradually reduced to four (anus level) and two (phasmid level). Lip region slightly narrower than the adjacent part of body, with six lips, the lateral ones smaller with six reduced labial and four prominent cephalic papillae. Amphidial apertures pore-like. Stoma reduced, with small cheilostom and elongate gymno-stegostom. Pharynx reduced with narrow corpus, slightly narrower isthmus, and pyriform basal bulb with reduced valves. Nerve ring surrounding the isthmus. Excretory pore located at metacorpus level. Hemizonid present, between NR and pharynx base. Cardia conoid. Deirids inconspicuous. Intestine bears a bacterial sac at its anterior part. Rectum long, almost straight, with very short cuticular part and elongate cellular part. Anus distinct. Genital primordium located equatorial. Tail conoid, tapering gradually with pointed terminus; cellular part longer than hyaline part, which comprises 28% to 39% of tail length; cellular-hyaline junction irregular. Phasmids located at 34% to 43% of tail length.

### Life cycle

*Steinernema africanum* n. sp. readily infests and develops in *G. mellonella* larvae. However, the development of *S. africanum* is unusually slow at 24°C compared to several other *Steinernema* species including *S. feltiae*, *S. weiseri*, *S. ischunanense*, *S. litorale*, *S. surkhetense*, *S. hermaphroditum*, *S. akhursti*, *S. cholashanense*, and *S. xueshanense*. We typically observe that *G. mellonella* larvae infested with 50 to 150 IJs of the above-mentioned species die within 2 d to 3 d, but insects take 5 d to 6 d to die when infested with *S. africanum*. Nematode adults of the first and second generations are found in insect cadavers within 5 d to 6 d and 8 d to 9 d, respectively, when infested by the above-mentioned species, while they are found after 8 d to 9 d and 11 d to 12 d, respectively, when infested with *S. africanum*. Pre-IJs emerge from insect cadavers after 12 d to 15 d upon infestation by the above-mentioned species, but only after 18 d to 21 d when infested by *S. africanum*.

### Type host and locality

The type hosts are unknown as the nematodes of this genus can be hosted by different insect species (Yan et al., 2016; Kajuga et al., 2018; Fallet et al., 2022) and were isolated from mixed soil samples by the *Galleria* baiting technique (White, 1927; Bedding and Akhurst, 1975). Briefly, *S. africanum* n. sp. RW14-M-C2b-1 and RW14-M-C2a-3 nematodes were isolated, using the “*Galleria* baiting” method, from soil samples collected in a banana, pumpkin, and sorghum intercrop in a valley in the Republic of Rwanda (GPS coordinates: 1°28’11.1”S 29°41’36.2”E; 1,865 m. s.

n. m.) (Yan et al., 2016). Cultures of this species are maintained in the Institute of Biology, University of Neuchatel (Switzerland), in the Rwanda Agriculture and Animal Resource Development Board (Rubona, Rwanda), and in CABI Swiss laboratories in Hungary.

### Type material

RW14-M-C2b-1 nematodes are the type material for *S. africanum* n. sp. Three slides of each stage, including first-generation adults (males and females), second-generation adults (males and females), and IJs, were deposited in the Nematology Collection of the Aquaculture Research Unit of the University of Limpopo, South Africa with the accession numbers ULRS-N1 to ULRS-N15. Additional specimens were deposited at the nematode collection of the Department of Animal Biology, Plant Biology and Ecology of the University of Jaén, Spain with the following accession numbers: RWA004-01 to RWA004-20 and RWA005-01 to RWA005-15. Slides will be made available upon reasonable request. Nematode cultures are maintained in the Institute of Biology, University of Neuchatel, Switzerland and in the Rwanda Agriculture and Animal Resource Development Board, Rubona, Rwanda.

### Etymology

The specific name refers to the continent where the species was isolated.

### Cross-hybridization experiments

No progeny was observed when *S. africanum* n. sp. (isolates RW14-M-C2b-1 or RW14-M-C2a-3 nematodes) were allowed to interact with specimens of *S. feltiae*, *S. ichnusae*, *S. litorale*, or *S. weiseri*. No progeny was observed in the single-female control plates. When *S. africanum* n. sp. (isolates RW14-M-C2b-1 or RW14-M-C2a-3 nematodes) were allowed to interact, fertile progeny was observed. Fertile progeny was also observed when all nematode strains were self-fertilized. Hence, *S. africanum* n. sp. (RW14-M-C2b-1 or RW14-M-C2a-3) are conspecific and reproductively isolated from closely related species such as *S. feltiae*, *S. ichnusae*, *S. litorale*, and *S. weiseri*.

### Diagnosis of Steinernema africanum n. sp

*Steinernema africanum* n. sp. adults have short stoma with rounded cheilorhabdia, pharynx robust with rounded basal bulb; males monorchid with ventrally curved spicules having lanceolate manubrium in the first generation and rounded manubrium in the second generation, gubernaculum fusiform in the first generation and anteriorly hook-like in the second generation, tail conoid and slightly ventrally curved with fine mucron in the first generation (34–46 mm, c = 25–34, c´ = 0.9–1.1, mucron = 4.0–5.3 mm) and with more robust mucron in the second generation (28– 46 mm, c = 26–36, c´ = 0.7–1.1, mucron = 5–10 mm); females didelphic–amphidelphic with shorter conoid tail bearing a fine mucron in the first generation (35–55 mm, c = 51–104, c´ = 0.7–1.0, mucron =3.7– 5.0 mm) and longer conoid, tail-lacking mucron in the second generation (40–60 mm, c = 17–51, c´ = 1.2– 2.3); and IJs with short body (0.69–0.80 mm), poorly developed pharynx (132–153 mm), H% (28–39), D% (79–105), and E% (135–290), lateral fields with eight longitudinal wings, and tail conoid-elongate (52– 72 mm, c = 10–15, c´ = 2.9–4.2).

### Morphological relationships of S. africanum n. sp. with other species

Based on morphological and morphometric traits, *S. africanum* n. sp. belongs to the “*feltiae*-clade.” Nematodes of this group are characterized by having third-stage IJs between 700 mm and 1,000 mm long. The lateral fields of IJs in the “*feltiae*-clade” are characterized by eight ridges arranged evenly in the mid-body region. *Steinernema africanum* n. sp., a member of the “*feltiae*clade,” presents several traits common to this group. Specifically, the IJs have a large body size (690–802 mm), and they have eight ridges in the mid-body region of the lateral field. Several of the morphometric traits of the IJs overlap with those of other species in the “*feltiae*-clade” (Tables 2 and 3).

**Table 2:** Comparative morphometrics of *Steinernema africanum* n. sp. IJs.

Species	Reference	L	BD	EP	NR	NL	TL	a	b	C	c'	D%	E%	H%
S. *africanum*	Present study	751	27	59	117	154	64	28	4.9	12	3.7	38	94	35
		(690‒802)	(25‒32)	(54‒68)	(87‒132)	(123‒167)	(52‒72)	(23‒30)	(4.3‒6.3)	(10‒15)	(2.9‒4.2)	(34‒46)	(79‒129)	(28‒39)
*S. akhursti*	Qiu et al. (2005)	812	33	59	90 (83‒95)	119	73	24	6.8	11	3.5	47	77	52
		(770‒835)	(33‒35)	(55‒60)		(115‒123)	(68‒75)	(23‒26)	(6.6‒7.2)	(10‒12)	(3.3‒3.7)	(45‒50)	(73‒86)	(49‒56)
*S*.	Ma et al. (2012)	843	30	62	87 (72‒97)	125	73	28	6.8	12	4.3	49	81	39
*cholashanense*		(727‒909)	(26‒35)	(59‒65)		(110‒138)	(60‒80)	(24‒34)	(6.1‒7.2)	(10‒14)	(3.5‒5.0)	(46‒53)	(76‒91)	(33‒47)
*S. citrae*	Stokwe et al. (2011)	754 (623‒849)	26 (23‒28)	56 (49‒64)	98 (83‒108)	125 (118‒137)	71 (63‒81)	30 (25‒34)	6.0 (5.1‒7.1)	15 (13‒17)	NA	44 (39‒58)	110 (85‒132)	43 (37‒50)
*S. feltiae*	Nguyen et al.	849	29	63	113	136	86	30	6.4	10	4.8	46	74	44
	(2007)	(766‒928)	(22‒32)	(58‒67)	(108‒117)	(130‒143)	(81‒89)	(27‒34)	(5.8‒6.8)	(9.4‒11)	(4.5‒5.1)	(44‒50)	(67‒81)	(37‒51)
*S. hebeiense*	Chen et al.	658	26	48	78 (73‒83)	107	66	26	6.2	10	NA	45	72	43
	(2006)	(610‒710)	(23‒28)	(43‒51)		(100‒111)	(63‒71)	(24‒28)	(5.7‒6.7)	(9.4‒11)		(40‒50)	(65‒80)	(32‒50)
*S. ichnusae*	Tarasco et al.	866	31	63	102	138	81	28	6.3	11 (9‒12)	4.6	46	77	48
	(2008)	(767‒969)	(27‒35)	(59‒68)	(94‒108)	(119‒148)	(76‒89)	(24‒32)	(5.6‒6.9)		(4.2‒5.1)	(42‒49)	(68‒83)	(44‒50)
*S. jollieti*	Spiridonov et al. (2004)	711	23	60	NA	123	68	31	5.7	10.5	4.5 (NA)	48	88 (NA)	55
		(625‒820)	(20‒28)	(53‒65)		(115‒135)	(60‒73)	(25‒34)	(4.9‒6.4)	(9‒12)		(46‒50)		(46‒60)
*S. kraussei*	Nguyen et al.	951	33	63	105	134	79	29 (NA)	7.1 (NA)	12.1 (NA)	3.9 (NA)	47 (NA)	80 (NA)	38
	(2007)	(797‒1,102)	(30‒36)	(50‒66)	(99‒111)	(119‒145)	(63‒86)							(35‒40)
*S. kushidai*	Mamiya (1998)	589	26	46	76 (70‒84)	111	50	23	5.3	11.7	NA	41	92 (NA)	NA
		(424‒662)	(22‒31)	(42‒50)		(106‒120)	(44‒59)	(19‒25)	(4.9‒5.9)	(10‒13)		(38‒44)		
*S. litorale*	Yoshida, (2004)	909	31	61	96	125	83	30	7.3	11	4.5	49	73	33 (NA)
		(834‒988)	(28‒33)	(54‒69)	(89‒104)	(114‒133)	(72‒91)	(27‒31)	(6.7‒7.9)	(10‒11.9)	(3.8‒5.4)	(44‒56)	(68‒84)	
*S. nguyeni*	Malan et al.	737	25	52	80 (74‒86)	110	67	29	6.7	11	4.3	48	79	27
	(2016)	(673‒796)	(22‒28)	(47‒58)		(101‒121)	(61‒73)	(27‒33)	(6.2‒7.4)	(10‒12)	(2.8‒4.8)	(43‒57)	(70‒86)	(20‒31)
*S. oregonese*	Liu and Berry, (1996)	980	34	66	NA	132	70	30	7.6 (6‒8)	14	4.7 (NA)	50	100	31
		(820‒1,110)	(28‒38)	(60‒72)		(116‒148)	(64‒78)	(24‒37)		(12‒16)		(40‒60)	(90‒110)	(30‒33)
*S. populi*	Tian et al. (2022)	1,095	36	77	106	149	64	30	7.4	17	2.8	52	121	35
		(973‒1,172)	(33‒41)	(70‒86)	(98‒113)	(134‒159)	(55‒72)	(24‒33)	(6.8‒8.5)	(15‒20)	(2.4‒3.3)	(47‒61)	(105‒140)	(26‒44)
*S. puntauvense*	Uribe-Lorfo	670	33	25	54 (46‒69)	94	54	20	6.1	12	NA	42	44	54
	et al. (2007)	(631‒728)	(31‒38)	(20‒30)		(81‒103)	(51‒59)	(17‒23)	(7.1‒7.9)	(11‒13)		(25‒50)	(35‒56)	(52‒55)
*S. sandneri*	Lis et al. (2021)	843	27	56	103	138	75	31	6.1	11.2	NA	40	74	34
		(708‒965)	(23‒32)	(44‒64)	(83‒118)	(123‒151)	(64‒86)	(27‒34)	(5.5‒6.9)	(11‒13)		(36‒45)	(63‒86)	(23‒40)
*S. sangi*	Phan et al. (2001)	753	35	52	91 (78‒97)	127	81	22	5.9	9.3	4.5 (NA)	40	62	49
		(704‒784)	(30‒40)	(46‒54)		(120‒138)	(76‒89)	(19‒25)	(5.6‒6.3)	(9‒10)		(36‒44)	(56‒70)	(44‒52)
*S*. *silvaticum*	Sturhan et al. (2005)	860	30	62	96	121	75	29	7.3	11.4	4.0	50	‒	46
		(670‒975)	(26‒35)	(51‒73)	(75‒109)	(100‒141)	(63‒86)	(23‒33)	(6.3‒7.7)	(10‒13)	(3.1‒4.9)	(46‒56)		(37‒53)
*S*. *tielingense*	Ma *et al*. (2012)	915	35	69	98	128	81	295	73	11	4	49	23	58
		(824‒979)	(32‒38)	(64‒73)	(90‒105)	(120‒135)	(74‒85)	(27‒31)	(67‒79)	(10‒12)	(3.5‒4.6)	(44‒56)	(68‒84)	(53‒64)
*S*. *texanum*	Nguyen et al. (2007)	756	30	59	92	115	73	25	6.5	10.4	3.3	51	81	59
		(732‒796)	(29‒34)	(52‒62)	(84‒102)	(111‒120)	(60‒79)	(22‒27)	(6.2‒7.0)	(10‒13)	(3.3‒4.6)	(46‒53)	(76‒88)	(53‒69)
*S*. *xinbinense*	Ma *et al*. (2012)	694	30	51	86 (75‒90)	116	73	24	6.1 (5‒7)	9.7	4.2 (3‒5)	44	71	35
		(635‒744)	(28‒31)	(46‒53)		(109‒125)	(65‒78)	(21‒25)		(8‒11)		(40‒47)	(65‒78)	(30‒42)
*S*. *xueshanense*	Mrácek et *al*.	860	30	67	91 (81‒96)	135	87	28	6.4	9.9	4.6	50	78	51
	(2009)	(768‒929)	(29‒33)	(60‒72)		(130‒143)	(80‒92)	(26‒32)	(5.8‒7.0)	(9‒11)	(3.8‒5.1)	(46‒52)	(70‒90)	(46‒55)
*S*. *weiseri*	Mrácek et al.	740	25	57	84 (72‒92)	113	60	29	6.6	12	3.7	51	95 (NA)	22
	(2003)	(586‒828)	(24‒29)	(43‒65)		(95‒119)	(49‒68)	(25‒33)	(5.7‒7.2)	(10‒14)	(3.2‒4.1)	(44‒55)		(18‒24)

All measurements are in μm (except ratios and percentages). BD, body diameter; EP, excretory pore; IJs, infective juveniles; NL, neck length; NR, nerve ring.

**Table 3:** Comparative morphometrics of first‒generation *Steinernema africanum* n. sp. males. All measurements are in micrometer (except ratios and percentages).

Species	L	BD	EP	NR	NL	TL	SL	GL	a	b	c	c'	D%	SW%	GS%
S. *africanum*	1,202	115	93	92	143	41	71	43	10	8	29	1.0	65	172	60
	(977‒1,400)	(65‒131)	(69‒109)	(79‒104)	(132‒147)	(34‒46)	(65‒76)	(32‒49)	(9‒12)	(7‒12)	(25‒34)	(0.9‒1.1)	(52‒74)	(144‒197)	(49‒68)
*S. akhursti*	1,589	131	102	136	182	35	90	64	NA	NA	NA	NA	56	180	71
	(1,350‒1,925)	(115‒150)	(93‒113)	(120‒163)	(168‒205)	(30‒40)	(85‒100)	(58‒68)					(52‒61)	(140‒200)	(65‒77)
*S*.	1,428	137	99	106	152	35	66	39	11	9.3	41	0.7	64	115	71
*cholashanense*	(1,070‒1,778)	(73‒204)	(75‒135)	(91‒126)	(135‒173)	(29‒43)	(60‒71)	(32‒45)	(9‒24)	(8‒11)	(36‒51)	(0.6‒0.9)	(50‒85)	(92‒144)	(61‒85)
*S. citrae*	1,154	103	81	106	139	25	65	44	NA	NA	NA	NA	58	198	68
	(1,028‒1,402)	(87‒113)	(64‒92)	(92‒119)	(123‒155)	(17‒31)	(57‒80)	(32‒59)					(47‒67)	(156‒233)	(48‒89)
*S. feltiae*	1,612 (1,414‒1,817)	75 (60‒90)	115 (110‒126)	69 (55‒87) a	170 (164‒180)	89 (37‒43)	70 (65‒77)	41 (34‒47)	11.5 (NA)	9.5 (NA)	41.3 (NA)	0.8 (NA)	60 (51‒64)	113 (99‒130)	59 (52‒61)
*S. hebeiense*	1,177	86	64	84 (78‒93)	126	30	57	46	14	9	39	0.8	51	140	80
	(1,036‒1,450)	(74‒98)	(58‒73)		(118‒132)	(24‒35)	(51‒63)	(38‒50)	(12‒17)	(8‒11)	(30‒49)	(0.6‒0.9)	(48‒59)	(120‒170)	(60‒90)
*S. ichnusae*	1,341	137	101	NA	165	40	66	44	22	8.2	34	0.8	62	139	67
	(1,151‒1,494)	(73‒204)	(94‒108)		(135‒173)	(33‒48)	(64‒67)	(43‒46)	(20‒29)	(7‒9)	(29‒39)	(0.8‒0.9)	(59‒65)	(120‒162)	(64‒69)
*S. jollieti*	1,662	115	98	NA	156	33	64	54	15	11	51	0.8 (NA)	64	145 (NA)	84 (NA)
	(1,296‒1,952)	(98‒135)	(83‒110)		(110‒168)	(24‒38)	(55‒70)	(45‒60)	(12‒19)	(8‒14)	(53‒86)		(53‒83)		
*S. kraussei*	1,400	128	81	105	153	39	49	33	11	9 (NA)	37 (NA)	0.9 (NA)	53 (NA)	110 (NA)	67 (NA)
	(1,200‒1,600)	(110‒144)	(73‒99)	(95‒122)	(137‒178	(36‒44)	(42‒53)	(29‒37)							
*S. kushidai*	1,400	97	84	129	167	33	63	44	NA	NA	NA	NA	51	150 (NA)	70 (NA)
	(1,200‒1,900)	(75‒156)	(71‒105)	(120‒137)	(156‒189)	(30‒40)	(48‒72)	(39‒60)					(42‒59)		
*S. litorale*	1,360	96	96	114	147	34	75	53	14	9.3	41	0.8	40	174	71
	(1,230‒1,514)	(82‒111)	(77‒107)	(94‒128)	(133‒163)	(26‒41)	(67‒89)	(44‒64)	(12‒16)	(8‒10)	(33‒56)	(0.6‒0.9)	(34‒56)	(154‒200)	(62‒81)
*S. nguyeni*	997	82	59	91	124	21	66	43	12	8	46	0.7	48	215	66
	(818‒1,171)	(58‒106)	(47‒71)	(70‒103)	(112‒144)	(18‒25)	(58‒75)	(30‒55)	(11‒15)	(7‒10)	(38‒53)	(0.6‒0.8)	(38‒57)	(185‒279)	(46‒81)
*S. oregonense*	1,680	138	112	111	154	29	71	56	NA	NA	NA	0.6 (NA)	73	151 (NA)	79 (NA)
	(1,560‒1,820)	(105‒161)	(95‒139)	(101‒133)	(139‒182)	(24‒32)	(65‒73)	(52‒59)					(64‒75)		
*S*.	1,591	119	94	115	140	33	77	34	NA	NA	NA	NA	67	170	65
*puntauvense*	(1,010‒1,931)	(101‒139)	(68‒114)	(104‒128)	(130‒159)	(28‒40)	(71‒81)	(30‒40)					(45‒85)	(140‒200)	(55‒75)
*S. sandneri*	1,461	155	80	126	157	41	60	44	10	9.3	37	NA	51	111	79
	(1,206‒1,635)	(124‒178)	(64‒92)	(112‒138)	(148‒170)	(35‒46)	(53‒65)	(39‒50)	(9‒11)	(8‒10)	(31‒42)		(42‒59)	(97‒127)	(61‒83)
*S. sangi*	1,774	159	82	126	166	32	63	40	NA	NA	NA	NA	49	150	60
	(1,440‒2,325)	(120‒225)	(67‒99)	(109‒166)	(150‒221)	(27‒42)	(58‒80)	(34‒46)					(42‒63)	(120‒160)	(50‒70)
S. *silvaticum*	1,090	65	79	119	142	34	51	37	17	7.7	34	1.0	60	155 (NA)	73 (NA)
	(975‒1,270)	(52‒78)	(71‒92)	(90‒126)	(116‒168)	(20‒47)	(42‒64)	(30‒43)	(14‒20)	(8‒9)	(24‒55)	(0.8‒1.4)	(45‒63)		
*S. tielingense*	1,778	129	114	112	160	26	88	62	11	11	70	0.5	71	191	73
	(1,430‒2,064)	(111‒159)	(94‒133)	(96‒132)	(145‒173)	(22‒33)	(79‒98)	(49‒70)	(11‒18)	(9‒13)	(57‒85)	(0.3‒0.6)	(64‒78)	(176‒212)	(59‒82)
*S. texanum*	1,296	99	90	104	135	23	60	45	NA	NA	NA	NA	67	157	75
	(1,197‒1,406)	(81‒116)	(79‒100)	(94‒114)	(123‒147)	(19‒30)	(55‒66)	(39‒53)					(58‒73)	(127‒203)	(62‒84)
*S. xinbinense*	1,265	103	68	106	149	37	56	35	12	8.5	34	0.9	45	137	63
	(1,133‒1,440)	(90‒126)	(57‒75)	(91‒120)	(138‒159)	(30‒41)	(49‒62)	(30‒41)	(11‒13)	(7‒9)	(31‒39)	(0.7‒1.0)	(41‒50)	(114‒156)	(54‒72)
*S*.	1,589	144	128	NA	160	38	76	49	NA	NA	NA	NA	80	152	64
*xueshanense*	(1,313‒2,040)	(97‒159)	(113‒137)		(151‒175)	(29‒48)	(66‒91)	(41‒60)					(73‒87)	(93‒172)	(58‒95)
*S. weiseri*	1,180	112	70	99	141	25	68	53	11	8	48	0.7	49	180	80
	(990‒1,395)	(84‒138)	(57‒84)	(94‒115)	(134‒154)	(19‒32)	(62‒72)	(46‒57)	(9‒12)	(7‒10)	(36‒64)	(0.6‒0.9)	(39‒60)	(150‒240)	(70‒85)

According to Aksary et al. (2020). BD, body diameter; EP, excretory pore; GL, gubernaculum length; NR, nerve ring; NL, neck length; SL, spicule length.

*Steinernema africanum* n. sp. IJs and first-generation adults are morphologically similar to *S. citrae*, *S. feltiae*, *S. ichnusae*, *S. litorale*, *S. nguyeni*, *S. weiseri*, *S. jollieti*, and *S. puntauvense*. The IJs of *S. africanum* n. sp. can be distinguished from the IJs of these latest species and other species of the “*feltiae*-clade” because the position of the NR is more posterior in *S. africanum* n. sp. and the stoma and pharynx regions are longer. In addition, *S. africanum* n. sp. IJs differ from *S. citrae* IJs in hyaline region occupying posterior (28%–39% vs 37%–50%) of tail length, hemizonid (present vs not observed), and cardia (conoid vs inconspicuous). *Steinernema africanum* n. sp., *S. feltiae, and S. ichnusae* IJs differ in tail length (52–72 μm vs 81–8 μm vs 76–89 μm), c´ (2.9–4.2 vs 4.5–5.1 vs 4.2–5.1), D% (34–46 vs 44–50 vs 42–49), hyaline region comprising (28–39% vs 37–51% vs 44–50%) of tail length, and E% (79–129 vs 67–81 vs 68–83). *Steinernema africanum* n. sp. and *S. litorale* IJs differ in body length (690–802 μm vs 834–988 μm), tail length (52–72 μm vs 72– 91 μm), b ratio (4.3–6.3 vs 6.7–7.9), and location of hemizonid (between NR and pharynx base vs basal bulb). *Steinernema africanum* n. sp. and *S. nguyeni* IJs differ in b ratio (4.3–6.3 vs 6.2–7.4), neck length (NL) (123–167 μm vs 101–121 μm), and hyaline region comprising (28–39% vs 20–31%) of tail length. *Steinernema africanum* n. sp. and *S. weiseri* IJs differ in deirids (inconspicuous vs located in center of lateral fields at level of pharyngeal bulb or slightly posterior), location of hemizonid (between NR and pharynx base vs at level of basal bulb or immediately posterior), hyaline region comprising (28–39% vs 18–24%) of tail length, and D% (34–46 vs 44–55). *Steinernema africanum* n. sp. and *S. jollieti* IJs differ in the number of structures of the lateral field at mid-body (eight vs six), D% (34–46 vs 46–50), and hyaline region comprising (28–39% vs 46–60%) of tail length. *Steinernema africanum* n. sp. and *S. puntauvense* IJs differ in hemizonid (present vs not observed), hyaline region comprising (28–39% vs 52–55%) of tail length, BD (25–32 μm vs 31–38 μm), distance from anterior region to EP (54–68 μm vs 20–30 μm), distance from anterior region to NR (87–132 μm vs 46–69 μm), a ratio (23–30 vs 17–23), and D% (79–129 vs 35–56) (Table 2).

**Table 4:** Comparative morphometrics of first‒generation *Steinernema africanum* n. sp. females. All measurements are in micrometer (except ratios and percentages).

Species	L	BD	EP	NR	NL	TL	a	b	c	c'	V	ABD	D%	Mucron
*S*. *africanum*	3,205	180	89	105	185	45	18	17	74	0.9	52	52	48	Present
	(2,469‒4,215)	(154‒194)	(67‒111)	(79‒130)	(170‒201)	(35‒55)	(13‒27)	(13‒24)	(51‒104)	(0.7‒1.0)	(50‒57)	(37‒70)	(36‒62)	
*S. akhursti*	7,283	239	126	164	239	49	29b (NA)	31b (NA)	141b	0.6b(NA)	51	86	53 (NA)	Present
	(5,625‒9,000)	(200‒270)	(113‒138)	(150‒175)	(213‒258)	(38‒63)			(NA)		(48‒53)	(68‒100)		
*S*.	4,692	255	129	190	196	57	13	25	83	0.8	53	77	50	Present
*cholashanense*	(3,232‒6,363)	(156‒332)	(111‒148)	(176‒223)	(181‒231)	(46‒70)	(13‒23)	(18‒32)	(62‒119)	(0.6‒1.0)	(50‒57)	(54‒105	(29‒65)	
*S. citrae*	3,087	175	75	151	215	44	NA	NA	NA	NA	54	62	37	Present
	(2,038‒4,019)	(137‒212)	(54‒90)	(130‒179)	(189‒220)	(33‒60)					(50‒59)	(43‒79)	(27‒46)	
*S. feltiae*	3,380	204	82	84 (70—97)a	237	52	17	14	65	1.0	56	52	46	Present
	(3,095‒3,774)	(170‒254)	(68‒97)a		(197‒304)	(39‒70)	(14‒20)	(12‒17)	(49‒88)	(0.7‒1.2)a	(44‒57)	(47‒62)	(40—54)a	
*S. hebeiense*	3,465	167	65	104	147	35	21	24	103	0.7	54	53	45	Absent
	(3,972‒4,254)	(142‒245)	(48‒95)	(88‒123)	(133‒158)	(25‒50)	(17‒25)	(21‒29)	(67‒129)	(0.5‒0.9)	(50‒57)	(45‒65)	(36‒66)	
*S. ichnusae*	5,514	269	126	NA	239	60	21	23	93	0.8	53	80	53	Present
	(4,547‒6,186)	(242‒323)	(106‒156)		(215‒262)	(51‒79)	(17‒24)	(21‒26)	(68‒113)	(0.6‒1.0)	(51‒57)	(70‒94)	(47‒63)	
*S. jollieti*	5,148	259	111	NA	214	43	20	26	128	NA	51	NA	52	Present
	(3,746‒6,030)	(219‒298)	(96‒136)		(184‒310)	(31‒55)	(15‒24)	(19‒31)	(72‒185)		(44‒56)			
*S. kraussei*	4,200	240	87	137	192	48	17	22	88	NA	54	45	45	Present
	(2,500‒5,400)	(153‒288)	(66‒99)	(127‒146)	(178‒205)	(33‒59)						(39‒50)		
*S. kushidai*	3,500	175	91	124	227	38	NA	NA	NA	NA	56	64	40	Absent
	(2,100‒4,700)	(54‒59)	(78‒105)	(111‒144)	(204‒255)	(30‒45)					(54‒59)	(54‒84)	(37‒46)	
*S. litorale*	4,462	191	88	146	196	39	23	23	117	0.6	56	62	45	Present
	(3,930‒5,048)	(175‒215)	(65‒105)	(130‒165)	(185‒213)	(25‒60)	(21‒26)	(20‒26)	(78‒157)	(0.5‒0.9)	(0.5‒0.9)	(55‒75)	(33‒57)	
*S. nguyeni*	4,775	178	72	113	169	32	21	22	119	0.7	56	178	43	Absent
	(2,290‒5,361)	(130‒216)	(49‒98)	(84‒139)	(137‒194)	(20‒67)	(15‒30)	(15‒30)	(53‒165)	(0.6‒1.1)	(52‒63)	(130‒216)	(30‒56)	
*S. oregonense*	5,200	242	103	147	210	37	NA	NA	NA	0.7 (NA)	52	56	49	Absent
	(4,400‒6,200)	(217‒268)	(217‒268)	(129‒162)	(186‒220)	(28‒46)					(46‒56)	(42‒79)	(43‒57)	
*S*.	6,198	198	70	135	192	49	NA	NA	NA	NA	53	76	37	Present
*puntauvense*	(3,687‒8,335)	(181‒221)	(51‒85)	(123‒146)	(141‒206)	(41‒66)					(51‒55)	(57‒102)	(25‒45)	
*S. sandneri*	4,628	210	84	147	185	147	22	25	102	NA	54	94	46	P‒esent
	(4,244‒5,014)	(181‒261)	(61‒102)	(132‒158)	(173‒194)	(32‒61)	(17‒25)	(24‒27)	(75‒140)		(49‒57)	(62‒122)	(36‒54)	
*S. sangi*	6,030	336	101	158	229	49	NA	NA	NA	NA	51	111	44	P‒esent
	(4,830‒7,200)	(270‒360)	(80‒121)	(140‒170)	(216‒240)	(936‒62)					(43‒530)	(84‒140)	(35‒51)	
S. *silvaticum*	2,150	116	69	113	146	45	19	15	49	1.3	52	35	47	Absent
	(1,520‒3,290)	(50‒175)	(50‒175)	(50‒175)	(121‒188)	(33‒79)	(15‒41)	(10‒18)	(34‒80)	(1.0‒1.8)	(44‒57)	(26‒53)	(33‒79)	
*S. texanum*	3,058	163	88	122	172	40	NA	NA	NA	NA	52	60	NA	Absent
	(2,720‒3,623)	(130‒202)	(78‒107)	(111‒135)	(160‒189)	(30‒52)					(50‒55)	(50‒71)		
*S. tielingense*	6,190	251	84	136	202	45	23	29	117	0.6	51	69	41	Absent
	(4,028‒8,538)	(200‒307)	(82‒103)	(111‒144)	(186‒263)	(40‒69)	(17‒32)	(21‒45)	(72‒158)	(0.5‒0.9)	(49‒54)	(56‒92)	(32‒49)	
*S. xinbinense*	4,037	176	80	126	186	40	22	22	106	0.6	52	61	40	Present
	(3,025‒5,121)	(159‒200)	(70‒87)	(106‒141)	(167‒192)	(30‒53)	(19‒25)	(17‒26)	(79‒123)	(0.5‒0.8)	(46‒57)	(50‒67)	(38‒45)	
*S*.	5,092	257	148	NA	230	55	NA	NA	NA	NA	54	84	NA	Present
*xueshanense*	(4,181‒8,181)	(182‒343)	(117‒148)		(196‒274)	(43‒66)					(52‒62)	(38‒72)		
*S. weiseri*	4,610	223	80	125	184	42	21	25	111	0.7	53	63	NA	Present
	(3,780‒5,940)	(202‒263)	(75‒86)	(108‒154)	(162‒226)	(38‒59)	(17‒29)	(22‒31)	(87‒156)	(0.5‒0.8)	(50‒58)	(51‒80)		

a According to Aksary et al. (2020). ^b^Calculated from Allotype BD, body diameter; EP, excretory pore; NR, nerve ring; NL, neck length.

First-generation males of *S. africanum* n. sp. differ from the males of *S. citrae* in lateral field (inconspicuous vs present in mid-body, with one narrow ridge) and tail length (34–46 μm vs 17–31 mm); from the males of *S. feltiae* in body length (0.98– 1.40 mm vs 1.41–1.81 mm*),* the position of the EP (69–109 μm vs 110–126 μm), NL (132–147 μm vs 164–180 μm), and SW% (144–197 vs 99–130); from the males of *S. ichnusae* in the presence of prominent mucron vs absent, spicule manubrium rhomboidal vs always oblongate, body size (0.98– 1.40mm vs 1.15–1.49 mm), and a ratio (9–12 vs 20–29); from the males of S. litorale in shape of spicule manubrium (rhomboidal vs oval to somewhat angular, rectangular), c (25–34 vs 33–56), D% (52–74 vs 34– 56), and GS% (49–68 vs 62–81); from the males of *S. nguyeni* in presence of amphidial apertures (small vs inconspicuous), lateral field inconspicuous vs with one ridge, the position of the EP (69–109 μm vs 47–71 μm), tail length (34–46 μm vs 18–25 μm), c (25–34 vs 38–53), and D% (52–74 vs 38–57); from the males of *S. weiseri* in the presence of mucron vs absent, spicule manubrium rhomboidal vs distinctly elongated, tail length (34–46 μm vs 19–32 μm), gubernaculum length (GL) (32–49 μm vs 46–57 μm), c (25–34 vs 36–64), and GS% (49–68 vs 70–85); from the males of *S. jollieti* in the presence of prominent mucron vs absent, shape of spicule manubrium (rhomboidal vs elongated), shape of gubernaculum (fusiform vs boat-shaped), deirids inconspicuous vs located posterior to level of pharyngo-intestinal junction, and a ratio (9–12 vs 12–19*);* from the males of *S. jollieti* in the distance of anterior end NR (79–104 μm vs 104– 128 μm), presence of spicule calomus (narrower vs inconspicuous), lamina with rostrum or retinaculum (present vs absent), and velum (scarcely developed velum not reaching the spicule tip vs extending from rostrum to spicule terminus) (Table 3).

### Nematode molecular characterization and phylogenetic relationships

**Figure 8:**
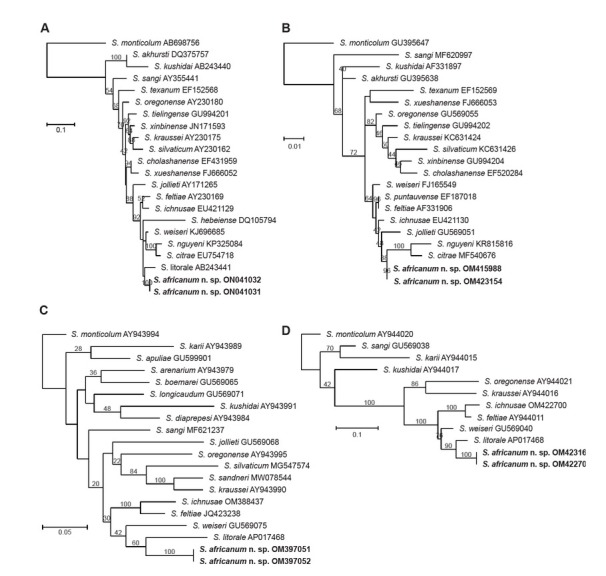
Phylogenetic relationships between the newly described *Steinernema africanum* n. sp. and other *Steinernema* species. Maximum-likelihood phylogenetic tree reconstructed from: **(A)** the nucleotide sequences of the ITS rRNA. A total of 705 nucleotide positions, flanked by primers 18S and 26S, were analyzed; **(B)** the nucleotide sequences of the D2–D3 expansion segments of the 28S rRNA. A total of 786 nucleotide positions, flanked by primers D2F and 536, were analyzed; **(C)** the nucleotide sequences of the COI gene. A total of 567 nucleotide positions, flanked by primers LCO-1490 and HCO-2198, were analyzed; and **(D)** the nucleotide sequences of the mitochondrial 12S rRNA gene. A total of 467 nucleotide positions, flanked by primers 505F and 506R, were analyzed. Numbers at nodes represent bootstrap values based on 100 replications. Bars represent average nucleotide substitutions per sequence position. NCBI accession numbers of the nucleotide sequences used for the analyses are shown next to the species names. COI, cytochrome oxidase subunit I; ITS, internal transcribed spacer; NCBI, National Center for Biotechnology Information.

**Figure S1:**
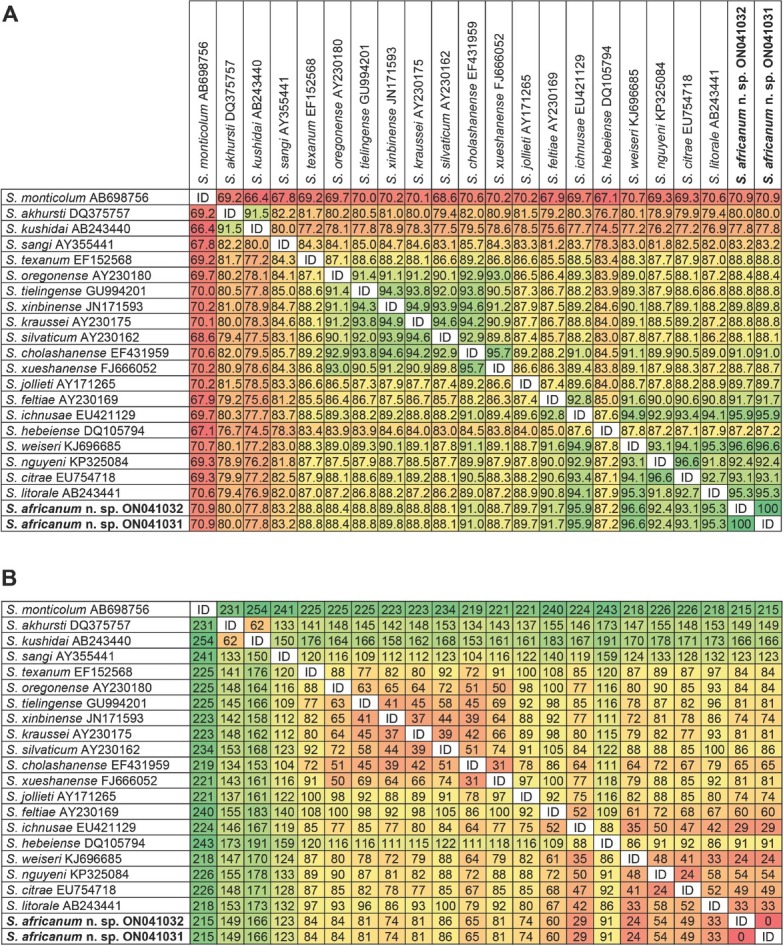
Pairwise comparisons of the ITS nucleotide sequences. **(A)** Sequence similarities (%). **(B)** Number of nucleotide differences (bp). A total of 808 nucleotide positions, flanked by primers 18S and 26S, were analyzed. NCBI accession numbers of the nucleotide sequences used for the analyses are shown next to the species names. ITS, internal transcribed spacer; NCBI, National Center for Biotechnology Information.

Phylogenetic reconstructions based on the nucleotide sequences of the D2–D3 expansion segments of the 28S rRNA, the ITS region of the rRNA, the mitochondrial 12S rRNA, and the COI show that *S. africanum* n. sp. belongs to the “*feltiae-*clade” (Fig. 8). Based on sequence similarities, *S. africanum* n. sp. is closely related to *S. citrae*, *S. ichnusae*, *S. litorale*, *S. nguyeni*, and *S. weiseri* (Figs. S1 and S2 in Supplementary Material). These species share between 92.4% and 95.9% and differ in 24 to 58 nucleotides with *S. africanum* n. sp. in the ITS sequences flanked by primers 18S and 26S (Fig. S1 in Supplementary Material). Less sequence similarities were observed between *S. africanum* n. sp. and all the other species of the “*feltiae*-clade,” supporting its novel taxonomic status (Figs. S1 and S2 in Supplementary Material).

### Symbiotic relationships

**Figure 9:**
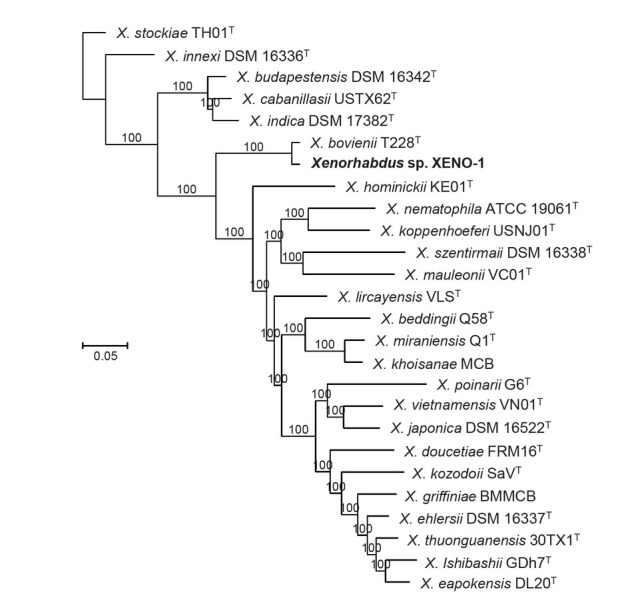
Phylogenetic relationships between the *Xenorhabdus* symbiont isolated from *Steinernema africanum* n. sp. and other *Xenorhabdus* species. Phylogenetic trees were built based on core genome sequences. A total of 1,719,910 nucleotide positions were used in the analyses. Numbers at the nodes represent SH-like branch supports. Bar represents 0.05 nucleotide substitutions per sequence position. Accession numbers of the genome sequences used for the reconstruction are shown in Table S1 in Supplementary Material.

Phylogenetic reconstructions based on whole genome sequences show that the bacterial symbiont isolated from *S. africanum* n. sp. RW14-M-C2b-1, named here XENO-1, is closely related to *X. bovienii* T228^T^ (Fig. 9). The dDDH value between *X. bovienii* T228^T^ and XENO-1 is 71.2%, suggesting that the symbiont of *S. africanum* n. sp. represents a novel subspecies within the *X. bovienii* species (Fig. S3 in Supplementary Material). This subspecies will be formally described elsewhere.

## Figures and Tables

**Table S1:** National Center for Biotechnology Information (NCBI) accession numbers of the sequences used in this study.

Strain	Genome
***Xenorhabdus sp.*** **XENO-1**	JAMGSK01
*X. beddingii* Q58^T^	MUBK01
*X. bovienii* T228^T^	JANAIF01
*X. budapestensis* DSM 16342^T^	NIBS01
*X. cabanillasii* USTX62^T^	QTUB01
*X. doucetiae* FRM16^T^	FO704550
*X. eapokensis* DL20^T^	MKGQ01
*X. ehlersii* DSM 16337^T^	NIBT01
*X. griffiniae* BMMCB	LDNM01
*X. hominickii* KE01^T^	NJAI01
*X. indica* DSM 17382T	NKHP01
*X. innexi DSM* 16336^T^	NIBU01
*X. ishibashi*i GDh7^T^	NJAK01
*X. japonica* DSM 16522^T^	FOVO01
*X. khoisanae* MCB	LFCV01
*X. koppenhoeferi* USNJ01^T^	FPBJ01
*X. kozodoii* SaV^T^	NJCX01
*X. lircayensis* VLS^T^	JACOII01
*X. mauleonii* VC01^T^	NITY01
*X. miraniensis* Q1^T^	NITZ01
*X. nematophila* ATCC 19061^T^	FN667742
*X. poinarii* G6^T^	FO704551
*X. stockiae* TH01^T^	NJAJ01
*X. szentirmaii* DSM 16338^T^	NIBV01
*X. thuongxuanensis* 30TX1^T^	MKGR01
*X. vietnamensis* VN01^T^	MUBJ01

**Figure S2:**
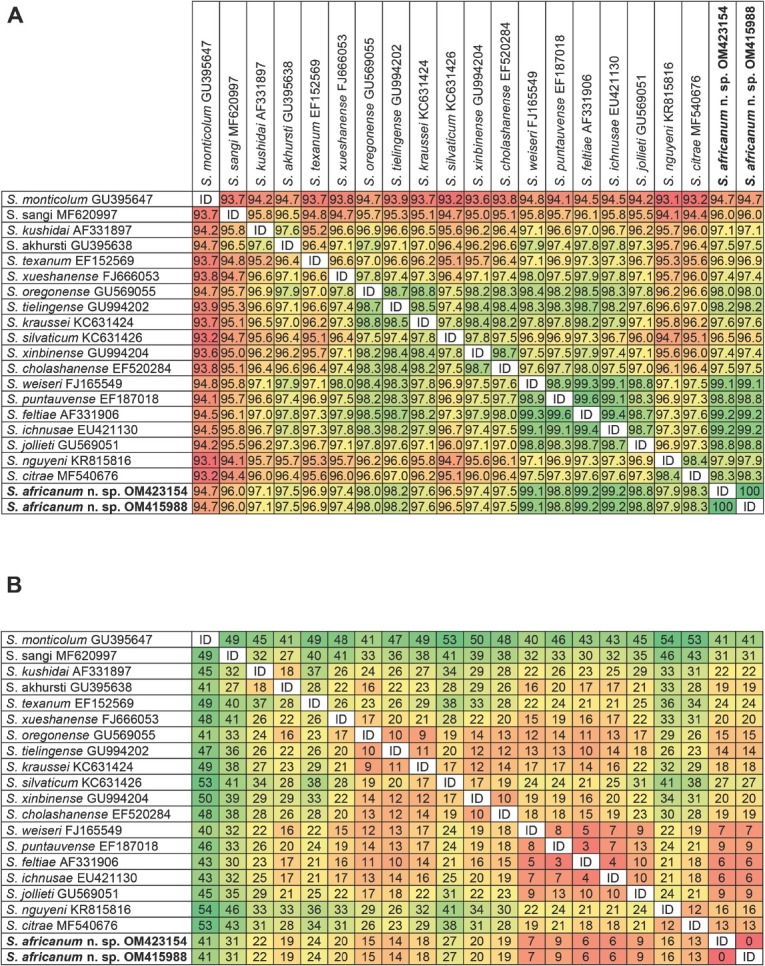
Pairwise comparisons of the nucleotide sequences of the D2–D3 expansion segments of the 28S rRNA. **(A)** Sequence similarities (%). **(B)** Number of nucleotide differences (bp). A total of 786 nucleotide positions, flanked by primers D2F and 536, were analyzed. NCBI accession numbers of the nucleotide sequences used for the analyses are shown next to the species names. NCBI, National Center for Biotechnology Information.

**Figure S3:**
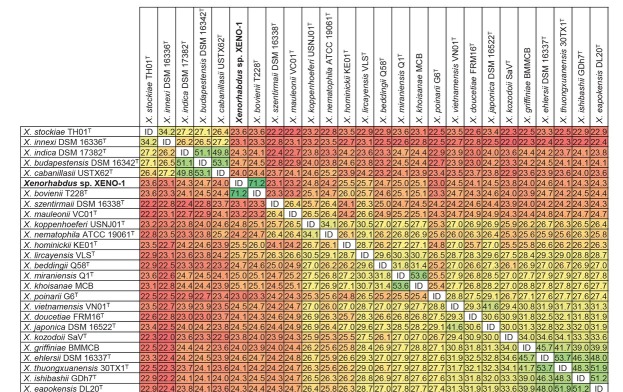
Pairwise comparison of dDDH scores (%) of *Xenorhabdus* strains. A total of 1,719,910 nucleotide positions were used in the analyses. Accession numbers of gene sequences used are shown in Table S1 in Supplementary Material. dDDH, digital DNA–DNA Hybridization.
